# New taxa of *Rhododendron tschonoskii* alliance (Ericaceae) from East Asia

**DOI:** 10.3897/phytokeys.134.38216

**Published:** 2019-10-23

**Authors:** Watanabe Yoichi, Tadashi Minamitani, Sang-Hun Oh, Atsushi J. Nagano, Harue Abe, Tomohisa Yukawa

**Affiliations:** 1 Graduate School of Horticulture, Chiba University, Matsudo 648, Matsudo, Chiba 271-8510, Japan Chiba University Matsudo Japan; 2 Tsunehisa 5-4-7, Miyazaki, Miyazaki 880-0913, Japan Unaffiliated Miyazaki Japan; 3 Department of Biology, Daejeon University, 62 Daehak-ro, Dong-gu, Daejeon 34520, South Korea Daejeon University Deajeon South Korea; 4 Faculty of Agriculture, Ryukoku University, Yokotani 1-5, Seta Oe-cho, Otsu, Shiga 520-2194, Japan Ryukoku University Otsu Japan; 5 Faculty of Agriculture, Niigata University, Koda 94-2, Sado, Niigata 952-2206, Japan Niigata University Sado Japan; 6 Tsukuba Botanical Garden, National Museum of Nature and Science, Amakubo 4-1-1, Tsukuba, Ibaraki 305-0005, Japan National Museum of Nature and Science Tsukuba Japan

**Keywords:** Ericaceae, new species, phylogeny, *
Rhododendron
*

## Abstract

Three new taxa, *Rhododendron sohayakiense* Y.Watan. & T.Yukawa (Ericaceae), and its two varieties, var. kiusianum Y.Watan., T.Yukawa & T.Minamitani and var. koreanum Y.Watan. & T.Yukawa are described and illustrated from Japan and South Korea. They can be distinguished from each other and from the other members of the *R. tschonoskii* alliance, i.e. *R. tschonoskii*, *R. tetramerum*, *R. trinerve* and *R. tsusiophyllum*, through their combination of leaf size, leaf morphologies including lateral nerves on abaxial leaf surface, corolla morphologies including number of corolla lobes, style length and anther form. Phylogenetic inferences based on chloroplast DNA and genome-wide sequences revealed that each of the three new taxa is monophyletic and they further form a clade. Distributions of the three taxa are also clearly separated from each other and also from the other members of the *R. tschonoskii* alliance.

## Introduction

The genus *Rhododendron* L. (Ericaceae) is morphologically diverse, comprising about 1,000 woody species (Chamberlain et al. 1996). The genus is mostly distributed across the Northern Hemisphere and members of subgenus Vireya extend into the Southern Hemisphere via the Indo-Australian Archipelago. The subgenus Tsutsusi is mostly found in East Asia (Yamazaki 1996; Kron and Powell 2009). Although most species of this subgenus occur in warm-temperate to subtropical regions, some are present in cold and alpine regions.

*Rhododendron tschonoskii* Maxim., *R. tetramerum* (Makino) Nakai, *R. trinerve* Franch. ex Boisser and *R. tsusiophyllum* Sugim. are closely related species placed within the subgenus Tsutsusi, which grow on exposed rocks or open sites in slopes and ridges on mountains. Among them, *R. tsusiophyllum* was originally described as a species of an independent genus *Tsusiophyllum tanakae* Maxim. (1870), because anthers of this species open through longitudinal slits while anthers of all other species in *Rhododendron* open through apical pores. The first taxonomic reappraisal of this group except *R. tsusiophyllum* was conducted by Takahashi (1975) in which he recognized the aforementioned four taxa at species rank. Subsequently, Yamazaki (1996) treated these two taxa as varieties of *R. tschonoskii*, i.e. R. tschonoskii var. tetramerum (Makino) Komatsu and R. tschonoskii var. trinerve (Franch. ex Boisser) Makino. In this study, we tentatively adopt Takahashi’s (1975) concept.

*Rhododendron tschonoskiisensu*Takahashi (1975) is widely distributed across the Japanese Archipelago and extends to the southern part of the Korean Peninsula. Despite the wide distribution, the species is absent from the central part of the Japanese Archipelago. As pointed out by Takahashi (1975) and Minamitani (1993), morphological characters of the species are distinct between eastern and western parts of the Japanese Archipelago. To evaluate these previous observations, we investigate morphological and macromolecular characters of *R. tschonoskii* and its related species by using the samples covering the entire species ranges.

## Methods

The morphological characters were observed and measured based on living materials in the field and herbarium specimens listed in the sections “Additional specimens examined”.

Samples for DNA analyses were collected from three individuals for *Rhododendron tschonoskii*, *R. tetramerum*, *R. trinerve*, *R. tsusiophyllum* and new entities, respectively. Three samples for each species and two entities and two samples for one entity (see Results and Discussion) were selected for covering entire range, and a holotype for each entity was included (Table 1). In addition, one individual for each species was collected from other relatives, i.e. *R. dilatatum*, *R. kaempferi*, *R. macrosepalum*, *R. reticulatum*, *R. serpyllifolium* and *R. tashiroi* belonging subgenus Tsutsusi (Kron and Powell 2009). For phylogenetic analysis, genomic DNA was extracted from silica-dried leaf samples using a DNeasy Plant mini kit (Qiagen, Hilden, Germany) after treatment with sorbitol buffer (Wagner et al. 1987). Five non-coding regions of chloroplast DNA (*trnL-F*, *trnL* intron, *trnS-G*, *trnG* intron and *rpl32-trnL*) were amplified and sequenced following the protocols described in Yoichi et al. (2017). The sequences were assembled using DNA Baser 4 (Heracle BioSoft, Pitești, Romania) and aligned using the MUSCLE algorithm implemented in MEGA 7 (Edgar 2004; Kumar et al. 2016).

**Table 1.** Locations of samples used for phylogenetic analyses.

Species	Code	Locality	Latitude / Longitude	Haplotype	Voucher
R. sohayakiense var. sohayakiense (Type 1)	Syk	Mt. Syakagadake, Nara, Japan	34.1145, 135.9020	H1	Y. Watanabe & K. Yukitoshi s.n.
	Miu	Mt. Miune, Tokushima, Japan	33.8398, 133.9877	H1	Y. Watanabe & T. Fukuda s.n.
	Ttj	Mt. Tsutsujyo, Ehime, Japan	33.7333, 133.1593	H1	Y. Watanabe & M. Takahashi Ttj02
R. sohayakiense var. kiusianum (Type 2)	Ici	Mt. Ichifusa, Kumamoto, Japan	32.3124, 131.1010	H2	Y. Watanabe Ici01
	Mks	Mt. Mukousaka, Kumamoto, Japan	32.5842, 131.1054	H2	Y. Watanabe Mks04
	Sob	Mt. Sobo, Miyazaki, Japan	32.8114, 131.3470	H3	Y. Watanabe & T. Oi s.n.
R. sohayakiense var. koreanum (Type 3)	Gom	Gonam, Jeollabuk-do, South Korea	35.4744, 127.5002	H4	Y. Watanabe, S. Hwang & N. Yun Gom01
	Wol	Mt. Wolbong, Gyeongsangnam-do, South Korea	35.7476, 127.7094	H4	Y. Watanabe, S. Hwang & N. Yun Wol01
*R. trinerve*	Snp	Mt. Sanpouiwa, Ishikawa, Japan	36.2586, 136.8441	H5	Y. Watanabe Snp02
	Sad	Sado Island, Niigata, Japan	37.9280, 138.4534	H5	H. Abe s.n.
	Iid	Mt. Iide, Niigata, Japan	37.9188, 139.5849	H6	Y. Wataanbe s.n.
*R. tetramerum*	Utk	Mt. Utsukushigahara, Nagano, Japan	36.2277, 138.0975	H7	Y. Watanabe s.n.
	Abe	Abe-touge pass, Shizuoka, Japan	35.3135, 138.3605	H7	Y. Watanabe Abe01
	Kmg	Mt. Kamagatake, Mie, Japan	35.0012, 136.4212	H7	Y. Watanabe & T. Oi s.n.
*R. tschonoskii*	Zao	Mt. Zao, Miyagi, Japan	38.1105, 140.4553	H8	Y. Watanabe Zao01
	Mus	Mt. Musadake, Hokkaido, Japan	43.6741, 144.8850	H8	Y. Watanabe s.n.
	Tor	Mt. Toraidake, Aomori, Japan	40.4505, 141.0100	H9	Y. Watanabe s.n.
*R. tsusiophyllum*	Hkn	Mt. Hakone-komagatake, Kanagawa, Japan	35.2235, 139.0233	H10	Y. Watanabe Hkn01
	Kdz	Kouzushima Island, Tokyo, Japan	34.13103, 139.0912	H11	H. Abe s.n.
	Tng	Mt. Tengu, Nagano, Japan	35.99188, 138.5689	H11	Y. Watanabe s.n.

Haplotype, Haplotype codes detected by chloroplast DNA sequences, corresponding to those in Fig. 3. Specimens of all of the analysed samples were deposited in TNS.

In addition, genome-wide SNPs were identified from two double digest restriction-site associated DNA (ddRAD) libraries using Peterson et al. (2012) protocol with some modifications. To fragment DNA sequences, 10 ng of genomic DNA was digested with *Eco*RI and *Bgl*II. Digestion and ligation were performed at 37 °C for 16 h in a 10 μL volume containing 20–40 ng of genomic DNA, 0.5 μL of each 10U/μL *Eco*RI and *Bgl*II enzyme (Takara, Kyoto, Japan), 1 μL of ×10 NEB buffer 2, 0.1 μL of ×100 BSA (New England Biolabs, Ipswich, USA), 0.4 μL of each 5 μM *Eco*RI and *Bgl*II adapter, 0.1 μL of 100 mM ATP and 0.5 μL of T4 DNA ligase (Enzymatics, Beverly, USA). The ligated product was purified with AMPure XP (Beckman Coulter, Brea, USA). The purified adaptor-ligated DNA was subsequently amplified by PCR. The PCR was performed in a 10 μL volume containing 2 μL of adaptor-ligated DNA, 2 μL of 5 μM index primer including 6-mer variable sequences for identifying different samples, 1 μL of 10 μM TruSeq universal primer, 5 μL of ×2 KAPA HiFi HotStart ReadyMix (KAPA Biosystems, Wilmington, USA). The PCR was performed with an initial denaturation for 4 mins at 94 °C, followed by 20 cycles of 10 s at 98 °C, 15 s at 65 °C and 15 s at 68 °C. The PCR products from different samples were pooled and purified again with AMPure XP. Fragments of 350–400 bp in the purified DNA solution were retrieved by electrophoresis using a 2.0% of E-Gel SizeSelect (Life Technologies, Carlsbad, USA). After quantity assessment using a QuantiFluor dsDNA System (Promega, Madison, USA) and quality assessment using an Agilent 2100 Bioanalyzer (Agilent Technologies, Santa Clara, USA), the libraries were sequenced with 51-bp single-end reads in two lanes of an Illumina HiSeq2000 (Illumina, San Diego, USA). After removing reads containing low-quality bases and adaptor sequences from the raw data using Trimmomatic v. 0.33 (Bolger et al. 2014), sequences with polymorphic SNPs were assembled by pyRAD v. 3.0 (Eaton 2014). Parameters for the assembly were set as follows: the minimum depth coverage for creating a cluster from reads was set to 6, the similarity threshold of clusters within and across individuals was set to 0.85, the maximum number of samples with shared heterozygous sites in a locus for filtering potential paralogs was set to 3, and polymorphic loci sequenced in more than half of the samples were finally exported as consensus sequences (Eaton 2014).

The phylogenetic relationships were inferred from two data sets, which were obtained from chloroplast DNA sequences and RAD-seq, based on the maximum likelihood method using RAxML v. 8.2.0 (Stamatakis 2014). In the analyses, the GTRGAMMA model was used as a substitution model, and node supports were assessed by bootstrap analysis with 1000 replicates. Phylogenetic relationships among individuals for *Rhododendron tschonoskii*, *R. tetramerum*, *R. trinerve*, *R. tsusiophyllum* and new entities based on RAD-seq were further evaluated by constructing a neighbor-net based on *p*-distance using SplitTree4 (Huson and Bryant 2006).

## Results and discussion

### Morphological differences

**Figure 1.**
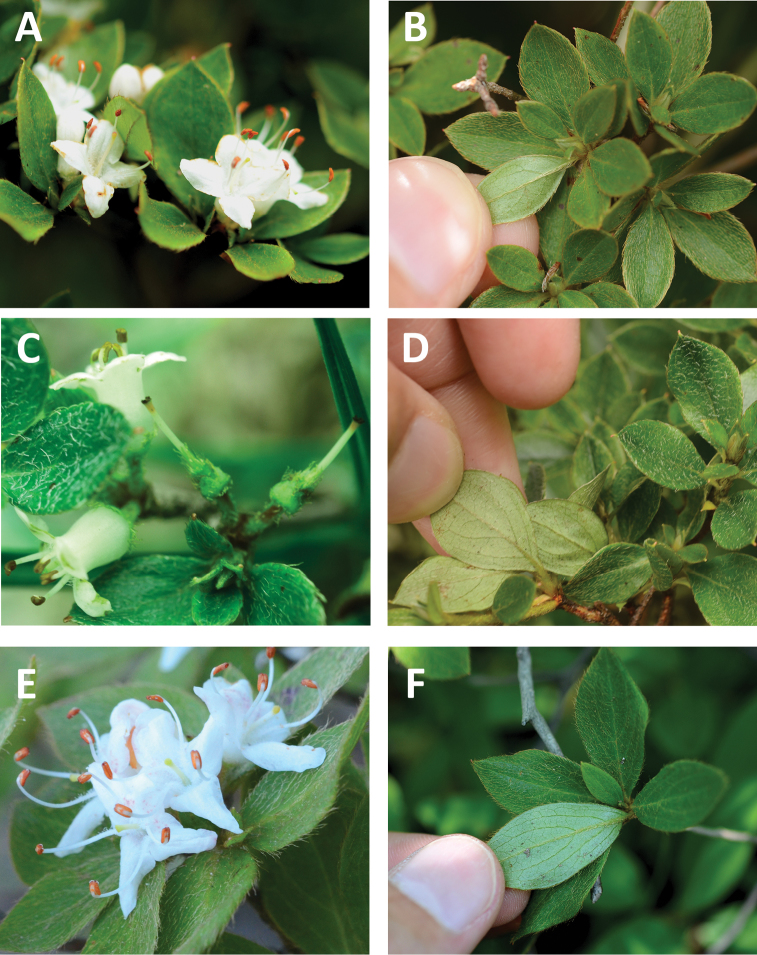
Photographs of flowers and leaves for the new taxa described in this study. **A, B**Rhododendron sohayakiense var. sohayakiense, Mt. Tsutsujo, Ehime Prefecture, Japan **C, D**Rhododendron sohayakiense var. kiusianum, Mt. Mukousaka, Kumamoto Prefecture, Japan **E, F**Rhododendron sohayakiense var. koreanum, Mt. Wolbong, Gyeongsangnam-do, South Korea. Photographs by Yoichi Watanabe.

**Figure 2.**
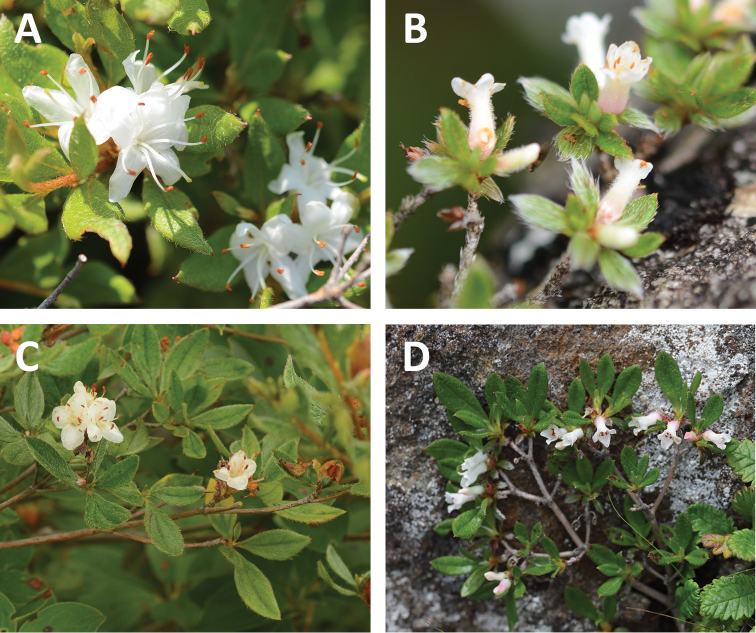
Photographs of flowers for the other members of *Rhododendron tschonoskii* alliance. **A***Rhododendron tschonoskii*, Mt. Bandai, Fukushima Prefecture, Japan **B***Rhododendron tsusiophyllum*, Mt. Kobushigatake, Yamanashi Prefecture, Japan **C***Rhododendron trinerve*, Mt. Sanpouiwa, Ishikawa Prefecture, Japan **D***Rhododendron tetramerum*, Mt. Yatsugatake, Nagano Prefecture, Japan. Photographs **A–C** by Yoichi Watanabe **D** courtesy of Osamu Takahashi.

We found three new entities (Types 1, 2 and 3) that have previously been included within *Rhododendron tschonoskii* (Fig. 2). However, corolla lobes for the three types, *R. trinerve* and *R. tetramerum* are tetramerous; contrastingly *R. tschonoskii* and *R. tsusiophyllum* are pentamerous. It is noteworthy to mention that the number of corolla lobes is sometimes variable within individuals. Further, they are distinguished from each other and from the other members of the *R. tschonoskii* alliance, i.e. *R. tschonoskii*, *R. tetramerum*, *R. trinerve* and *R. tsusiophyllum* through their leaf size, leaf morphologies including lateral nerves on abaxial leaf surface, corolla morphologies and style length (Table 2, Figs 1, 2). Leaf sizes of Types 1, 2 and *R. tschonoskii* are medium (5–30 mm long), while Type 3 and *R. trinerve* are large (10–50 mm long). Lateral nerves on the abaxial surface of the leaf are pinnate but obscure for Type 1, while 1–3 pairs of pinnate lateral nerves are raised for Types 2 and 3. Corolla tube lengths of Types 1, 3, *R. tschonoskii* and *R. trinerve* are short (2–4 mm long), while Type 2, *R. tetramerum* and *R. tsusiophyllum* are long (3.5–7 mm long). Styles of Types 1, 3, *R. tschonoskii* and *R. trinerve* are longer than the corolla tube and exserted from the corolla, while those of Type 2, *R. tetramerum* and *R. tsusiophyllum* are similar or shorter than the corolla tube and included within the corolla.

The three types share the following combination of characters as commonly derived character states, which can be distinguished from the other members of the *R. tschonoskii* alliance. The corolla form of the three types are tubular-funnelform and corolla lobes are tetramerous; in addition, lateral nerves on abaxial leaf surface are raised or obscure raised, and not prominent, such as *R. trinerve*.

**Table 2.** Diagnostic characters among the *Rhododendron tschonoskii* alliance.

Character	R. sohayakiense var. sohayakiense (Type 1)	R. sohayakiense var. kiusianum (Type 2)	R. sohayakiense var. koreanum (Type 3)	*R. tschonoskii*	*R. tsusiophyllum*	*R. trinerve*	*R. tetramerum*
Leaf length (mm)	10–20	10–20	10–35	10–25	5–13	15–50	5–20
Leaf width (mm)	4–7	5–10	5–15	5–12	2–5	5–20	5–10
Nerve on abaxial surface of leaf	pinnate 2–3 pairs, obscurely raised	pinnate 2–3 pairs, raised	pinnate 1–3 pairs, raised	nervules reticulate	nervules obscurely reticulate	pinnate 1–2 pairs, prominent, nervules reticulate	nervules obscurely reticulate
Hair of leaf	densely strigose on adaxial surface, glabrous or sparsely strigose on abaxial surface	densely strigose on adaxial surface, sparsely strigose on abaxial surface	densely strigose on adaxial surface, sparsely strigose on abaxial surface	strigose on both surfaces	densely strigose on adaxial surface, glabrous on abaxial surface	strigose on both surfaces	densely strigose on adaxial surface, glabrous or sparsely strigose on abaxial surface
Corolla form	tubular-funnelform	tubular-funnelform	tubular-funnelform	tubular-funnelform	tubiform	tubular-funnelform	tubiform
Number of corolla lobes	4	4	4	5	5	4	4
Corolla tube length (mm)	2–3	3–4	2–3	2–4	5–7	2–4	3.5–4.5
Corolla lobe length (mm)	3–5	2–5	3–5	4–6	ca. 2	4–6	2–3
Style length (mm)	4–10	3–4	5–6	6–13	4–5	3–7	2.5–3.5
Style condition	exserted	included	exserted	exserted	included	exserted	included
Anther	opening by apical pores	opening by apical pores	opening by apical pores	opening by apical pores	opening by longitudinal slits	opening by apical pores	opening by apical pores
Distribution	Japan: Honshu (Kii Peninsula) and Shikoku	Japan: Kyushu	South Korea: Gyeongsang and Jeolla provinces	Japan: Honshu (Kanto and Tohoku districts) and Hokkaido. Russia: Kunashir Island	Japan: Honshu (Chubu and Kanto districts)	Japan: Honshu (Kinki, Chubu, Kanto and Tohoku districts)	Japan: Honshu (Kinki, Chubu and Kanto districts)

### Phylogenetic relationships

**Figure 3.**
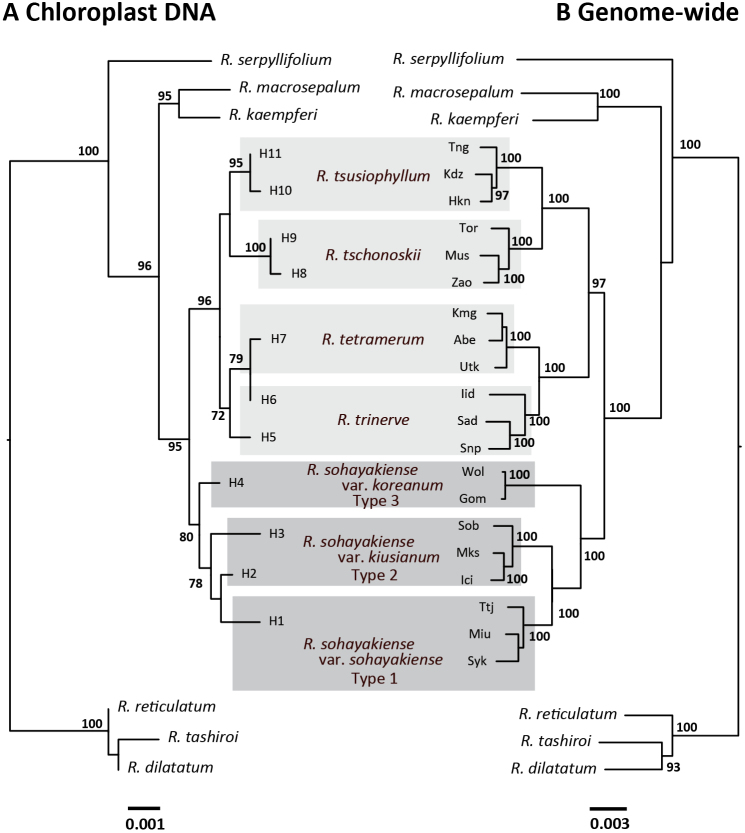
Comparative phylogenies for **A** haplotypes based on chloroplast DNA sequences and **B** genotypes based on restriction site associated DNA sequences (RAD-seq). Bootstrap probabilities (> 70%) are shown above nodes. Gray boxes indicate three new taxa described in this study and light gray boxes indicate the other members of the *Rhododendron tschonoskii* alliance.

**Figure 4.**
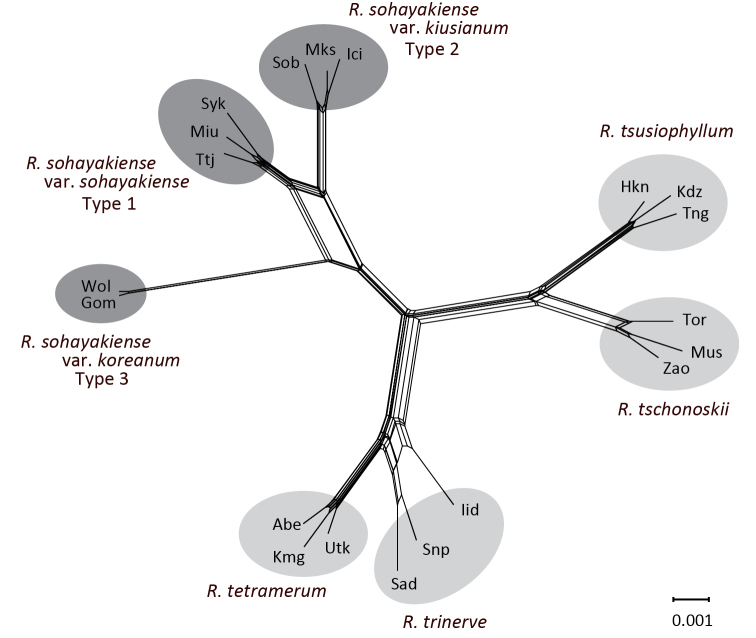
Neighbor-net for members of the *Rhododendron tschonoskii* alliance reconstructed from *p*-distance among individuals based on RAD-seq.

Phylogenetic relationships of the *R. tschonoskii* alliance based on chloroplast DNA sequences (2,847 bp with 76 polymorphic sites) and genome-wide sequences (RAD-seq, 316,455 bp with 37266 SNPs) were almost concordant including outgroup species (Fig. 3). The *R. tschonoskii* alliance formed a monophyletic group (95% for chloroplast DNA and 100% for RAD-seq). The chloroplast DNA sequences identified four haplotypes from the three types, Types 1 and 3 had one haplotype respectively and Type 2 had two haplotypes. Although the monophyly of the clade comprising Types 1, 2 and 3 was supported with high bootstrap probability (80%), the monophyly of two haplotypes in Type 2 was not supported. The monophyly of the clade comprising Types 1, 2 and 3 and the monophyly of each type were supported with the highest bootstrap probabilities (100%) based on RAD-seq. The neighbor-net for a data set, which included only the *R. tschonoskii* alliance, identified three groups corresponding to Types 1, 2 and 3, which can be clearly distinguished from each other (Fig. 4).

Thus the three types can be distinguished from each other and also from the other members of the *R. tschonoskii* alliance. The results indicate that the three types should be treated as different taxa but these are more closely related than the others in the *R. tschonoskii* alliance. Since we confirmed the independent state of the three types from morphological and phylogenetic characteristics, we hereby describe them as a new species and its two varieties.

### Taxonomic treatments

#### Rhododendron sohayakiense

Taxon classificationPlantaeEricalesEricaceae

Y.Watan. & T.Yukawa sp. nov.

E02531F7-0782-5853-A31F-7A414B89866E

urn:lsid:ipni.org:names:77202559-1

Figs 1 , 5 , 6

##### Diagnosis.

This species is similar to *Rhododendron tschonoskii* Maxim, but is distinguishable through its 4 corolla lobes and its pinnate nerves on the adaxial leaf surface.

##### Type.

**JAPAN. Shikoku**: Ehime Pref., Kumakogen Town, Mt. Tsutsujo-yama, 33°44'00.01"N, 133°09'33.40"E, on ridge of the mountain, 1800 m, 20 July 2016 (fl), Y. Watanabe and M. Takahashi Ttj02 (holotype TNS; isotypes TNS, KYO).

##### Additional specimens examined.

**JAPAN. Kii Peninsula, Honshu**: Nara Pref., Yoshino County, Kamikitayama Village, Mt. Oodaigahara, Daijyagura, 1500 m, 23 Aug 1956 (fr), G. Murata 10133 (KYO); Nara Pref., Yoshino County, Kamikitayama Village, Mt. Oodaigahara, Daijyagura, 1600 m, 18 Jul 2012 (fl), K. Yamawaki 4869 (KYO); Nara Pref., Yoshino County, Shimokitayama Village, Mt. Kujyaku, 1800 m, 17 Jul 1954 (fl), G. Murata & T. Shimizu 104 (KYO); **Shikoku**: Tokushima Pref., Miyoshi County, Higashiiya Village, Mt. Tsurugi, 1950 m, 25 Jul 1986 (fl), G. Murata et al. 45946 (KYO); Tokushima Pref., Miyoshi County, Higashiiya Village, Mt. Tsurugi, 1700m, 22 Oct 2012 (fr), Y. Katayama 32 (KYO); Tokushima Pref., Miyoshi County, Nishiiyayama Village, Mt. Nakatsu, 1400 m, 10 Aug 1954, G. Murata 7728 (KYO); Kochi Pref., Nagaoka County, Otoyo Village, Mt. Kajigamori, 1200 m, 22 Aug 1964, G. Murata 18671 (KYO); Ehime Pref., Kamiukena County, Kumakougen Town, Mt. Ishizuchi, between Dogamori and summit, 1600–1980 m, 27 Jul 1983 (fl), G. Murata 44754 (KYO); Ehime Pref., Saijyo City, Mt Ishizuchi, between starting point and summit, 1400–1850 m, 17 Jul 1992 (fl), T. Minamitani 43440 (TNS); Ehime Pref., Niihama City, Mt. Douzanmine, 1300 m, 15 Jul 1980 (fl), K. Tsuchiya 491 (KYO); Ehime Pref., Uma County, Mt. Higashiakaisi and Mt. Futatsudake, 1600 m, 8 Sep 1961 (fr), G. Murata 14993 (KYO); Ehime Pref, Niihama City, Mt. Higashiakaishi, 33°52'31.03"N, 133°22'26.31"E, 1700 m, 15 Jul 2017 (fl), Y. Watanabe Hga03 (TNS).

##### Description.

Much branched semi-evergreen shrubs 1–1.5 m tall. Branchlets and petioles with dense appressed flattened brownish strigose hairs. Spring leaves scattered or crowded on upper branchlets; petioles 0.5–1 mm long; blade thick chartaceous, oblong, 10-20 mm long (at maximum within each individual), 4–7 mm wide, apex acute and terminating in a gland, base acute, adaxial surface green, abaxial surface pale green, densely strigose on adaxial surface, glabrous or sparsely strigose on abaxial surface without midrib; midrib prominent abaxially; lateral nerves pinnate, 2–3 paired, obscure raised abaxially. Summer leaves oblanceolate, 5–10 mm long, 1–6 mm wide, densely strigose on both surfaces. Flower buds terminal, single, broadly ovoid, acute, ca. 2 mm long, 2 mm wide; scales widely ovate, densely strigose on upper outer surface. Inflorescences umbel-like, 2–4 flowers. Pedicel 2–4 mm long at flowering, densely appressed hirsute. Calyx saucer-shaped, ca. 1.5 mm in diam., densely strigose, shallowly 4-lobed; lobes semiorbiculate, ca. 0.5 mm long. Corolla white, no blotches, openly tubular-funnelform, 8–12 mm long and wide, dissected 1/2 corolla length into 4 lobes; tube 2–3 mm long, ca. 2 mm wide, glabrous outside, pilose on upper inside; lobes elliptic to oblong, rounded, 3–5 mm long, 2–4 mm wide. Stamens 4, subequal, 5–8 mm long, exserted; filaments densely pilose on lower three-quarters; anthers yellow, oblong, ca. 1 mm long. Ovary ovoid, densely soft strigose, ca. 1.5 mm. Style 4–10 mm long, exserted, glabrous. Capsule ovoid, 2–5 mm long, 2–3 mm wide, densely strigose.

##### Distribution.

JAPAN: Honshu (Kii Peninsula), Shikoku.

##### Ecology.

The plants inhabit sunny places and grow on mountain ridges and slopes at altitudes over 1000 m above sea level. In such places, there are few trees and established communities of shrubs and dwarf bamboos (*Sasa* sp.). Flowering specimens have been collected from July to August; fruiting specimens have been collected from October to November. Bumblebees are frequent visitors to the flowers, suggesting that they are pollinators of the species.

##### Etymology.

The specific epithet refers to ‘Sohayaki’ a floristic region in Japan that covers Kii Peninsula of Honshu, Shikoku and Kyushu (Koidzumi 1931), where the new species is distributed.

**Figure 5.**
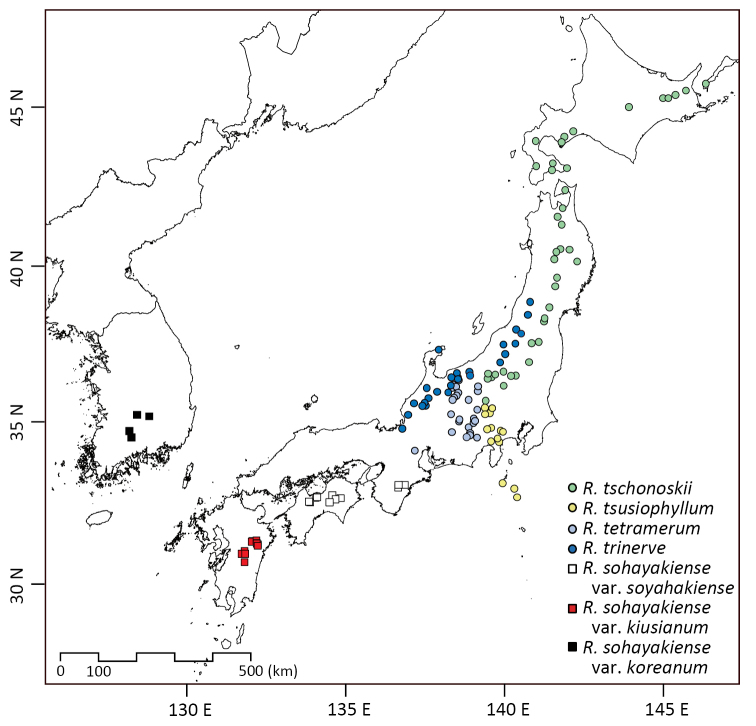
Distribution of the *Rhododendron tschonoskii* alliance. Circles and squares showed locations of herbarium specimens (KYO, TNS).

**Figure 6.**
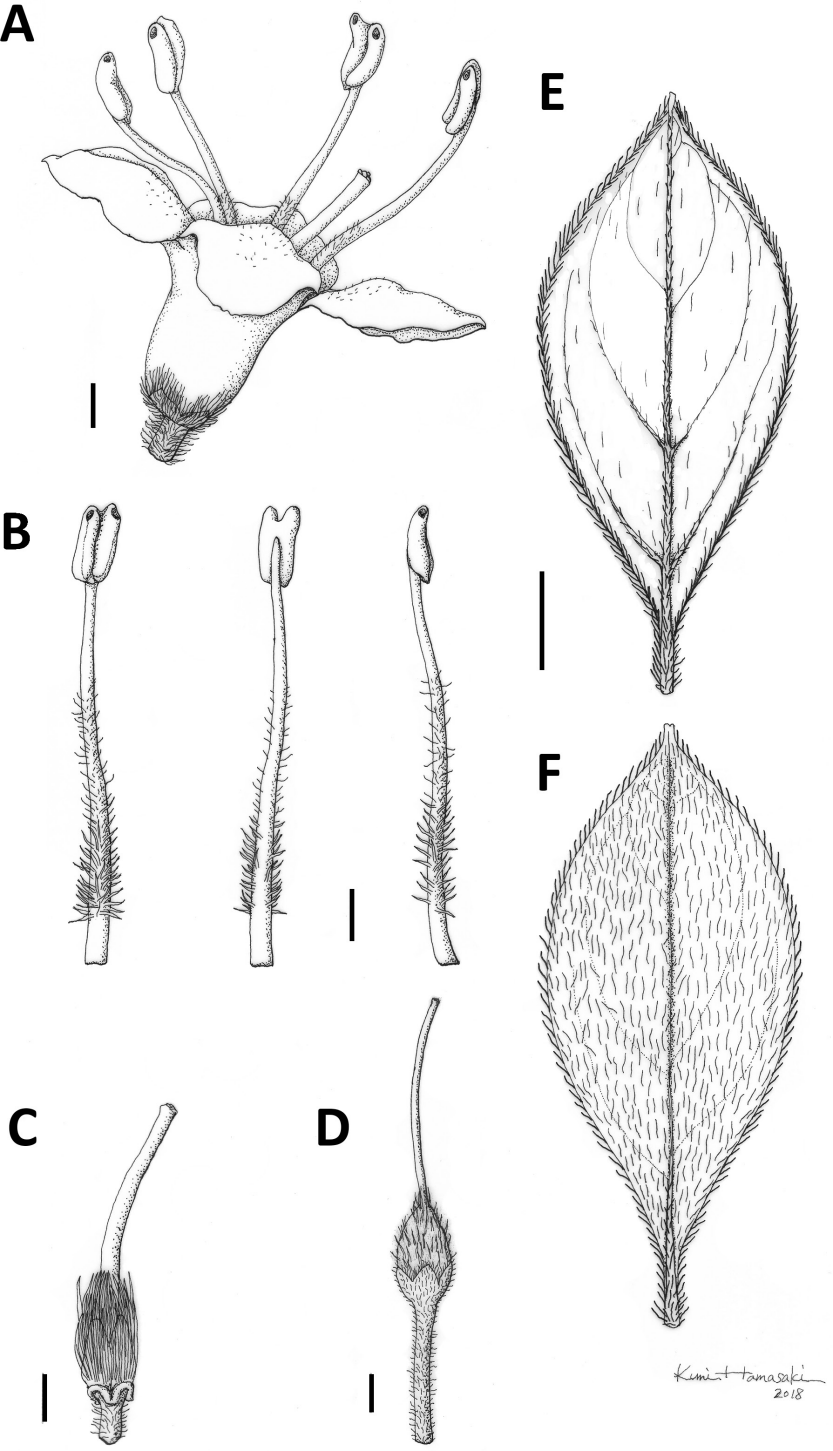
Rhododendron sohayakiense var. sohayakiense. **A** Flower, side view **B** Stamen **C** Pistil **D** Fruit **E** Abaxial and **F** adaxial sides of leaf. Scale bars: 1 mm (**A–D**); 3 mm (**E, F**). Drawings by Kumi Hamasaki from *Y. Watanabe & M. Takahashi Ttj02* (holotype, TNS).

#### Rhododendron sohayakiense var. kiusianum

Taxon classificationPlantaeEricalesEricaceae

Y.Watan., T.Yukawa & T.Minamitani var. nov.

A166957A-EECE-57F3-A441-4DFD07C5B821

urn:lsid:ipni.org:names:77202565-1

Figs 1 , 5 , 7

##### Diagnosis.

This variety is similar to Rhododendron sohayakiense var. sohayakiense Y. Watan. & T. Yukawa and R. sohayakiense var. koreanum Y. Watan. & T. Yukawa, but is distinguishable through its longer corolla tube and its shorter style included within the corolla. Further, it differs from the former through its raised lateral nerves on the abaxial leaf surface and from the latter through its small leaf size.

##### Type.

**JAPAN. Kyushu**: Kumamoto Pref., Kamimashiki County, Yamato Village, Mt. Mukosaka, 32°35'03.22"N, 131°06'19.30"E, on rocky ridge of the mountain, 1500 m, 23 July 2016 (fl), Y. Watanabe Mks04 (holotype: TNS; isotypes: TNS, KYO).

##### Additional specimens examined.

**JAPAN. Kyushu**: Ooita Pref., Mt. Katamuki, 2 Aug 1921 (fl), Z. Tashiro (KYO); Miyazaki Pref., Higashi-Usuki County, Shiiba Village, Mt. Eboshi, 1690 m, 18 Jul 2007 (fl), T. Minamitani (TNS); Miyazaki Pref., Higashi-Usuki County, Shiiba Village, Mt. Ougi, 1200–1661 m, 7 Jul 1994 (fl), T. Minamitani 049666 (TNS); Miyazaki Pref., Nishi-Usuki County, Hinokage Town, Mt. Goyou, 1570 m, 11 Aug 1990 (fr), T. Minamitani (TNS); Miyazaki Pref., Nishi-Usuki County, Hinokage Town, Mt. Katamuki, 1500 m, 25 Aug 1970 (fr), T. Minamitani (TNS); Miyazaki Pref., Nishi-Usuki County, Takachiho Town, Mt. Tengu, 1700 m, 24 Jun 1993 (fl), T. Minamitani (TNS); Miyazaki Pref., Nishi-Usuki County, Takachiho Town, Mt. Tengu, 1600–1700 m, 16 Aug 1992, T. Minamitani 44119 (TNS); Miyazaki Pref., Nishi-Usuki County, Takachiho Town, Mt. Shoji, 1600–1700 m, 16 Aug 1992, T. Minamitani 44128 (TNS); Miyazaki Pref., Nishi-Usuki County, Gokase Town, Mt. Mukosaka, Tsutsujigaoka, 1570 m, 12 Jul 1993 (fl), T. Minamitani (TNS); Miyazaki Pref., Nishi-Usuki County, Gokase Town, Mt. Mukosaka, Kita, 1550 m, 24 Sep 1996 (fl, fr), T. Minamitani B-T-052838 (TNS); Miyazaki Pref., Higashi-Usuki County, Kitakata Town, Mt. Hoko, 1100–1200 m, 26 Jun 1994 (fl), T. Minamitani (TNS); Miyazaki Pref., Higashi-Usuki County, Kitakata Town, Mt. Hoko, 1270 m, 23 Sep 1989 (fr), T. Minamitani (TNS); Miyazaki Pref., Nobeoka City, Kitakata, Mt. Ohkue, Kozumidaki, 1340 m, 6 Aug 1992 (fr), T. Minamitani (TNS); Kumamoto Pref., Kuma County, Mt. Ichifusa, 1700 m, 6 Aug 1960 (fl), M. Tagawa & K. Iwatsuki 3676 (KYO); Kumamoto Pref., Kuma County, Yunomae Town, Mt. Ichifusa, 26 Jul 1992 (fl), T. Minamitani 43478 (TNS); Kumamoto Pref., Kuma County, Mizukami Village, Mt. Ichifusa, 32°18'44.65"N, 131°06'03.50"E, on summit of the mountain, 1600 m, 24 July 2016 (fl), Y. Watanabe Ici02 (TNS).

##### Description.

Spring leaves scattered or crowded on upper branchlets; petioles 0.5–1 mm long; blade thick chartaceous, oblong-ovate, 10–20 mm long (at maximum within each individual), 5–10 mm wide, apex acute and terminating in a gland, base acute, strigose on both surfaces; midrib prominent abaxially; lateral nerves pinnate, 2–3 paired, raised abaxially. Summer leaves oblanceolate, 3–10 mm long, 1–5 mm wide, densely strigose on both surfaces. Calyx saucer-shaped, ca. 1.5 mm in diam., densely soft strigose, shallowly 4-lobed; lobes semiorbiculate, ca. 0.5 mm long. Corolla white, no blotches, tubular-funnelform, 7–13 mm long and wide, 4 lobes; tube 3–4 mm long, ca. 3 mm wide, glabrous outside, pilose on upper inside; lobes elliptic to oblong, rounded, 2–5 mm long, ca. 2 mm wide. Stamens 4, irregular, 3–5 mm long, as long as or shorter than corolla; filaments densely pilose on lower half; anthers yellow, oblong, ca. 1 mm long. Ovary ovoid, densely soft strigose, ca. 1.5 mm. Style 3–4 mm long, glabrous, shorter than corolla. Capsule ovoid, 3–4 mm long, 2.5 mm wide, densely strigose.

##### Distribution.

JAPAN: Kyushu.

##### Ecology.

The plants inhabit sunny and rocky mountain ridges and slopes at altitudes over 1000 m above sea level. Flowering specimens have been collected from July to August; fruiting specimens have been collected from October to November.

##### Etymology.

The specific epithet refers to ‘Kyushu’ where the new variety is distributed.

##### Note.

Although the style of this variety is included within the corolla, this part is exserted from the corolla in individuals from Mt. Ichifusa.

**Figure 7.**
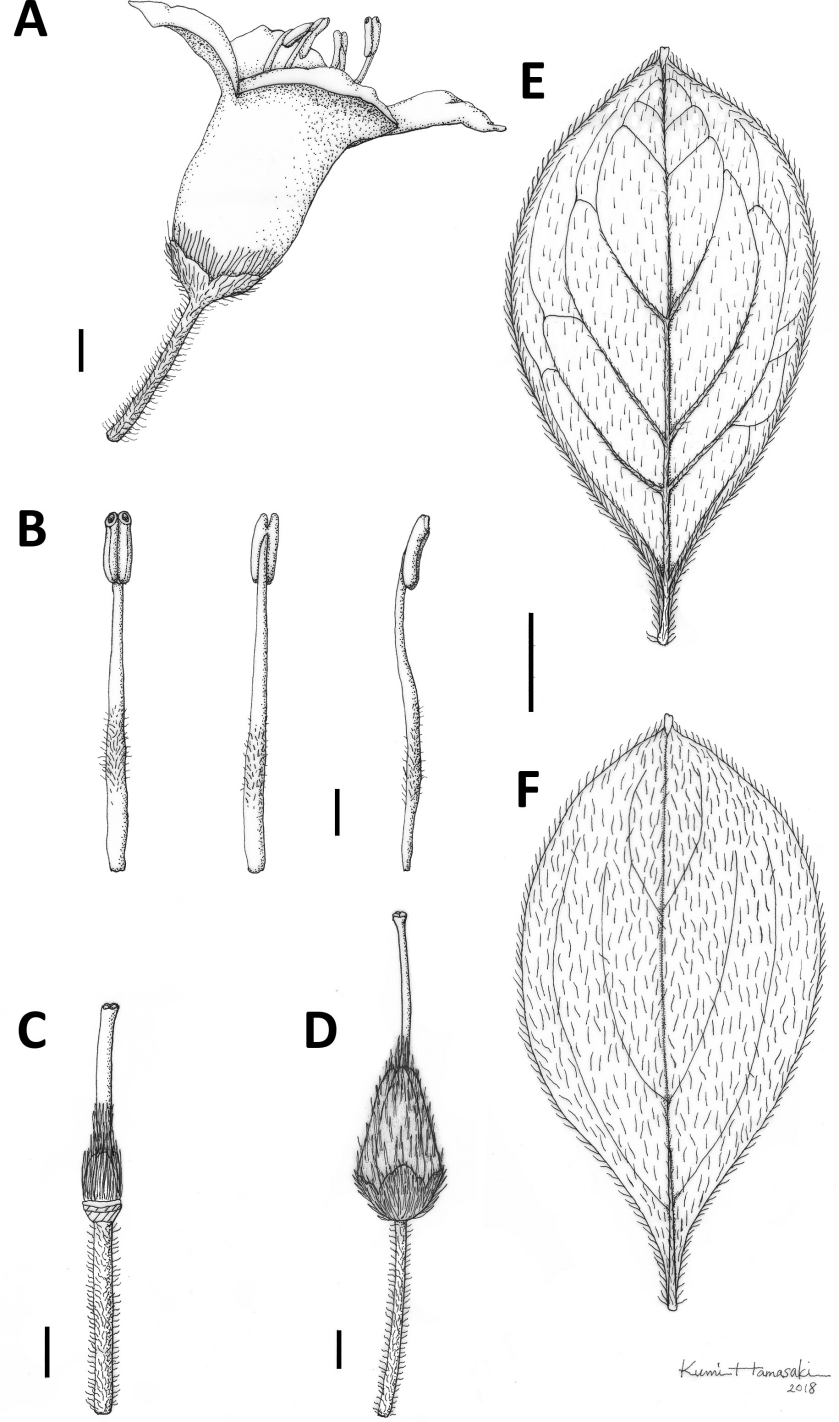
Rhododendron sohayakiense var. kiusianum. **A** Flower, side view **B** Stamen **C** Pistil **D** Fruit **E** Abaxial and **F** adaxial sides of leaf. Scale bars: 1 mm (**A–D**); 3 mm (**E, F**). Drawings by Kumi Hamasaki from *Y. Watanabe Mks04* (holotype, TNS).

#### Rhododendron sohayakiense var. koreanum

Taxon classificationPlantaeEricalesEricaceae

Y.Watan. & T.Yukawa var. nov.

1C3CC705-8553-5CC2-825F-C4FA68396F3D

urn:lsid:ipni.org:names:77202566-1

Figs 1 , 5 , 8

##### Diagnosis.

This variety is similar to Rhododendron sohayakiense var. sohayakiense Y. Watan. & T. Yukawa and R. sohayakiense var. kiusianum Y. Watan., T. Yukawa & T. Minamitani, but is distinguished by its large leaf size. Further, it differs from the former through its raised lateral nerves on the abaxial leaf surface and from the latter through its shorter corolla tube and by its longer style exserted from the corolla.

##### Type.

**SOUTH KOREA. Gyeongsangnam-do**: Hamyang County, Mt. Wolbong, 35°44'51.31"N, 127°42'33.99"E, on slope of the mountain, 1000 m, 15 June 2017 (fl), Y. Watanabe, S. Hwang and N. Yun Wol06 (holotype: TNS; isotypes: TNS, KB).

##### Additional specimens examined.

**SOUTH KOREA. Jeollabuk-do**: Namwon City, Mt. Gonam, 35°28'27.74"N, 127°30'00.82"E, 800 m, 15 Jun 2017 (fl), Y. Watanabe, S. Hwang and N. Yun Gom01 (TNS); Mt. Jirisan, Banyabong peak, 1700 m, 20 Aug 1982 (fr), T. Yamazaki & F. Yamazaki 3294 (KYO); **Gyeongsangnam-do**: Mt. Gayasan, 23 Aug 1935 (fr), G. Koidzumi (KYO).

##### Description.

Spring leaves scattered or crowded on upper branchlets; petioles 0.5–1 mm long; blade thick chartaceous, oblong, 10–35 mm long (25–35 mm long at maximum within each individual), 5–15 mm wide, apex acute and terminating in a gland, base acute, strigose on both surfaces; midrib prominent abaxially; lateral nerves pinnate, 1–3 paired, raised abaxially. Summer leaves oblanceolate, 6–20 mm long, 1–8 mm wide, densely strigose on both surfaces. Calyx saucer-shaped, ca. 1.5 mm in diam., densely soft strigose, shallowly 4-lobed; lobes semiorbiculate, ca. 0.5 mm long. Corolla white, no blotches, openly tubular-funnelform, 8–12 mm long and wide, dissected 1/2 corolla length into 4 lobes; tube 2–3 mm long, ca. 2 mm wide, glabrous outside, pilose on upper inside; lobes elliptic to oblong, rounded, 3–5 mm long, 2–4 mm wide. Stamens 4, subequal, 6–9 mm long, exserted; filaments densely pilose on lower three-quarters; anthers yellow, oblong, ca. 1 mm long. Ovary ovoid, densely soft strigose, ca. 2 mm. Style 5–6 mm long, exserted, glabrous. Capsule ovoid, 3–4 mm long, 2.5 mm wide, densely strigose.

##### Distribution.

SOUTH KOREA: Jeollabuk-do, Gyeongsangnam-do.

##### Ecology.

The plants inhabit mountain ridges and slopes at altitudes over 800 m above sea level. Flowering specimens have been collected from June to August; fruiting specimens have been collected from October. Honeybees are frequent visitors to the flowers, suggesting that they are pollinators of the variety.

##### Etymology.

The specific epithet refers to ‘Korea’ where the new variety is distributed.

**Figure 8.**
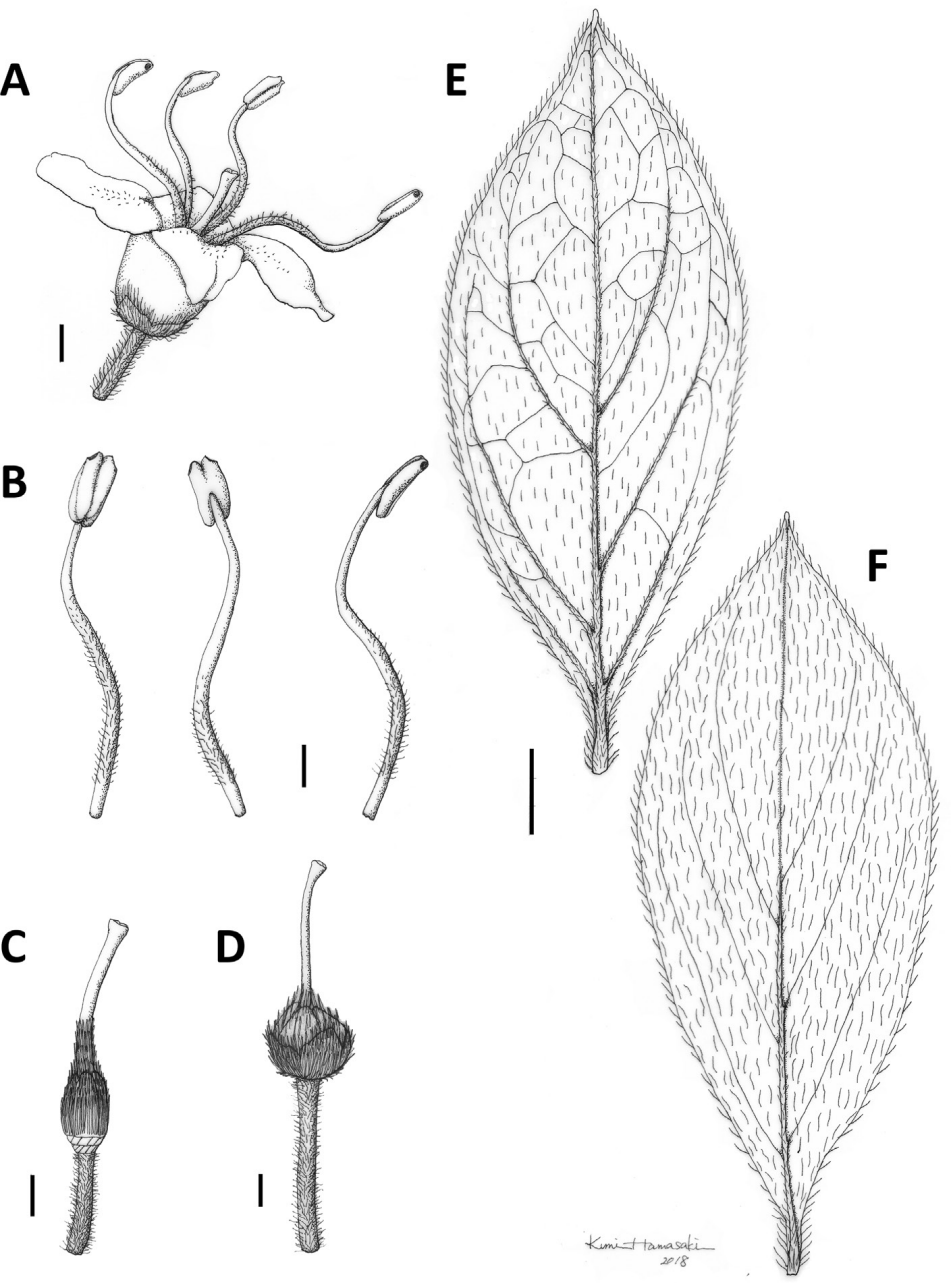
Rhododendron sohayakiense var. koreanum. **A** Flower, side view **B** Stamen **C** Pistil **D** Fruit **E** Abaxial and **F** adaxial sides of leaf. Scale bars: 1 mm (**A–D**); 3 mm (**E, F**). Drawings by Kumi Hamasaki from *Y. Watanabe*, *S. Hwang and N. Yun Wol01* (holotype, TNS).

### Key to the *Rhododendron tschonoskii* alliance, expanded from Yamazaki (1996)

**Table:** 

1	Corolla 4 lobes	**2**
–	Corolla 5 lobes	**6**
2	Corolla tubiform	***R. tetramerum***
–	Corolla tubular-funnelform	**3**
3	Spring leaves 25–50 mm long at maximum within each individual	**4**
–	Spring leaves 10–20 mm long at maximum within each individual	**5**
4	Lateral nerves of spring leaf 1 paired, prominently raised abaxially, grooved adaxially, nervules reticulate	***R. trinerve***
–	Lateral nerves of spring leaf 1–3 paired, raised abaxially, not grooved adaxially	**R. sohayakiense var. koreanum**
5	Style 4–10 mm long, exserted from corolla; lateral nerves of spring leaf obscurely raised	**R. sohayakiense var. sohayakiense**
–	Style 3–4 mm long, included within corolla; lateral nerves of spring leaf raised	**R. sohayakiense var. kiusianum**
6	Corolla tubular-funnelform, tube 2–4 mm long; style 6–13 mm long, exserted from corolla	***R. tschonoskii***
–	Corolla tubiform, tube 5–7 mm long; style 4–5 mm long, included within corolla	***R. tsusiophyllum***

## Data accessibility

DNA sequences of chloroplast DNA haplotypes reported in this study were deposited in GenBank under accession numbers; LC499847–LC499863 for *trnL-F*, LC499864–LC499880 for *trnL* intron, LC499830–LC499846 for *trnS-G*, LC499813–LC499829 for *trnG* intron, LC499796–LC499812 for *rpl32-trnL*. Genotype data for RAD-seq were deposited in Dryad: https://doi.org/10.5061/dryad.5tm6680.

## Supplementary Material

XML Treatment for Rhododendron sohayakiense

XML Treatment for Rhododendron sohayakiense var. kiusianum

XML Treatment for Rhododendron sohayakiense var. koreanum
